# ICU-acquired weakness: should medical sovereignty belong to any specialist?

**DOI:** 10.1186/s13054-017-1923-7

**Published:** 2018-01-04

**Authors:** Domenico Intiso

**Affiliations:** Unit of Neuro-Rehabilitation, Hospital IRCCS “Casa Sollievo della Sofferenza”, Viale dei Cappuccini, 71013 San Giovanni Rotondo, FG Italy

**Keywords:** ICU-acquired weakness, Critical illness polyneuropathy, Intensivist, Multi-specialist, Recovery

## Abstract

ICU-acquired weakness (ICUAW), including critical illness polyneuropathy, critical illness myopathy, and critical illness polyneuropathy and myopathy, is a frequent disabling disorder in ICU subjects. Research has predominantly been performed by intensivists, whose efforts have permitted the diagnosis of ICUAW early during an ICU stay and understanding of several of the pathophysiological and clinical aspects of this disorder. Despite important progress, the therapeutic strategies are unsatisfactory and issues such as functional outcomes and long-term recovery remain unclear. Studies involving multiple specialists should be planned to better differentiate the ICUAW types and provide proper functional outcome measures and follow-up. A more strict collaboration among specialists interested in ICUAW, in particular physiatrists, is desirable to plan proper care pathways after ICU discharge and to better meet the health needs of subjects with ICUAW.

## Background

ICU-acquired weakness (ICUAW) is a frequent disabling disorder that can occur in ICU subjects. Given that the disorder can involve the muscular and peripheral nervous systems, many definitions have been suggested including critical illness polyneuropathy (CIP), critical illness myopathy (CIM), and critical illness polyneuropathy and myopathy (CIPNM), but until now no definition has obtained unanimous consensus. With regard to this issue, ICUAW is proposed to overcome nomenclature classification problems [1] even if CIPNM is also broadly accepted. Although clinical assessment of muscle weakness using the Medical Research Council (MRC) score can quantify strength impairment, differentiation of the ICUAW types is not possible on the basis of the clinical picture, and electromyography (EMG) remains the hallmark in diagnosing and differentiating ICUAW types, particularly in volitional subjects. Since the first description by Bolton et al. [2], ICU specialists have carried out a number of investigations that have provided important progress in understanding several aspects of ICUAW, including its pathogenic mechanisms as well as electrophysiological [3] and histological pictures [4]. Recently, a reduction in the sodium channel subtype Nav1.6 was found on the sural nerve of ICU patients by Li et al. [5], who also observed production of antibodies against all three major sodium channels (Nav1.6, Nav1.8, Nav1.9) which have a major role in the initiation and conduction of action potentials. Furthermore, experimental animal model studies in rats have demonstrated a hyperpolarized shift in the voltage dependence of sodium channels [6] and impaired Ca^2+^ release, which induce muscle membrane inexcitability and muscle weakness [7]. These findings support pioneering studies hypothesizing that the pathological mechanism responsible for CIM could be due to muscle membrane inexcitability [8]. On the other hand, although great progress has been made by ICU specialists, several areas of uncertainty persist that should be addressed in future research [9]. Among these, pharmacological therapy to prevent and better manage this disorder has remained unsatisfactory. Indeed, despite the number of therapeutic interventions investigated, including antioxidant and nutritional agents, corticosteroids, and intravenous immunoglobulins, only intensive insulin therapy has been demonstrated to produce some benefit [10]. Likewise, nonpharmacological treatments have been ineffective, apart from early physical therapy which has been found to reduce the duration of mechanical ventilation [10]. Other important issues that should be addressed concern the functional outcomes and long-term recovery of ICUAW subjects.

## ICU-acquired weakness: clinical course and recovery

ICUAW is a major cause of chronically impaired motor function that can affect activities of daily living and quality of life; therefore, proper prognosis as well as previewing the clinical course and recovery represent crucial aspects in the management of ICUAW subjects. A number of studies have investigated functional outcomes and disability in subjects who survive a critical illness. ICUAW subjects and, in particular, older adults who survive critical illness suffer physical and cognitive declines that result in disabilities at greater rates than hospitalized, noncritically ill and community-dwelling older adults [11]. Differentiating between ICUAW types could be essential when considering prognosis and recovery since the outcomes of subjects suffering from ICUAW have generally been correlated with ICUAW type. Indeed, it has been reported that subjects with the CIM type have a better prognosis than those suffering from CIP or CIP/CIM and achieve full recovery within 6–12 months after ICU discharge [12, 13]. Nonetheless, few reports have documented that CIP and CIM can have different outcomes, and the impact on long-term physical function, particularly of CIP and CIP/CIM, is unclear. As mentioned previously, EMG is the benchmark for differentiating ICUAW types, but the examination results might be uncertain and doubtful in the early stages of the disease. It well known that proper and accurate EMG requires collaboration of the subject, a condition which is difficult to attain in all patients during an ICU stay. Likewise, this condition can also be observed in subjects with a severe disability and limited consciousness after ICU discharge. Therefore, to better differentiate the CIM type, an electrophysiological study (EPS) has been proposed that defines direct muscle stimulation (dmCMAP) and evaluates the CMAP amplitude to calculate the ratio between nerve stimulation (neCMAP) and direct muscle stimulation CMAP (neCMAP/dmCMAP) [3, 14, 15]. Although dmCMAP could represent a valid strategy for differentiating the CIM type, the test is time-consuming and is not widely used in clinical practice. Studies concerning the outcomes and long-term recovery of subjects on the basis of differentiated ICUAW types are scant. Furthermore, such studies have severe limitations, including small sample sizes, lack of proper functional evaluation measurements, and short duration of follow-up. The only systematic review of the long-term recovery of subjects with ICUAW types reported that the mean duration of follow-up was 3–6 months and did not generally exceed 2 years [16].

Further important aspect to consider is that most studies addressing functional outcomes in subjects suffering from ICUAW types investigated patients with this disorder, regardless of the primary cause of admission to the ICU. Actually, researchers enrolled subjects whose primary disorder on ICU admission was predominantly due to nonneurological causes, such as general and cardiac surgery, respiratory failure, and sepsis [4, 17–19], depending on the ICU type. The course of and what happens in subjects with acquired brain damage who develop ICUAW remain unknown. No study has considered the outcome of ICUAW in subjects whose primary cause of ICU admission was a central nervous impairment, such as severe brain damage, although these patients represent a conspicuous share of ICU admissions. The previously mentioned review investigating the outcomes of differentiated ICUAW types considered studies that also enrolled subjects in whom the primary cause of ICU admission was severe brain injury [13, 16]. However, the authors did not specify whether subjects who had coexisting acquired brain damage experienced different outcomes. Our group has recently reported that one-third of ICU subjects with severe acquired brain injury (sABI) admitted to a dedicated rehabilitative setting suffered from ICUAW. Although the functional recovery of these patients improved after rehabilitation, they had poorer outcomes and significantly longer rehabilitative stays than sABI subjects without ICUAW [20]. Furthermore, in a previous study, our group observed that ICU subjects with a combination of sABI and ICUAW showed reduced quality of life and greater disability compared to patients without ICUAW at 5 years of follow-up, and those with CIP/CIM showed poorer functional improvement [21]. ICUAW has been detected at a rate of 90.9% in subjects in a vegetative or minimally responsive state following sABI [22]. This finding raises several questions regarding the relationship between primary causes of ICU admission and ICUAW, length of ICU stay, risk factors, and prevention.

The combination of brain damage and ICUAW has not been considered previously to avoid biasing the results [23–25]. On the other hand, in studies enrolling subjects with brain damage, it was not possible to exclude other reasons due to cultural tendencies and the interests of the specialists who carried out the investigations. In this regard, ICUAW is considered to be a clinical condition that may represent the extreme end of a spectrum of weakness that can follow any serious illness regardless of care location [26].

A recent report by ICU specialists has recommended that age, premorbid ICU condition, and functionality should be considered when evaluating ICUAW outcomes [9] because these factors could affect the functional trajectory and strongly influence the post-ICU functional status. However, when investigating ICUAW recovery, further aspects should also be considered, such as the effect of the ICUAW types, effect of the primary cause of ICU admission, role of each disorder in impairments and disability, and rehabilitation interventions in particular. To date, no randomized clinical trials have been conducted to test whether physical therapy (PT) and specific rehabilitative interventions improve the outcomes and activities of daily live for people with ICUAW [27]. Likewise, apart from a study by Novak et al. [28], who reported that patients with ICUAW achieved a significant improvement in activities and participation after rehabilitation, no data have been published on the effect of rehabilitation on quality of life and participation of these patients when considering the International Classification of Functioning, Disability and Health (ICF) domains [27].

## Managing ICU-acquired weakness

Latronico [29] recently admonished ICU specialists, stating they should pay attention to CIP and avoid the risk of this disorder becoming a “no man’s land” (i.e., a territory without the sovereignty of any specialist). Furthermore, he exhorted them to consider patients with CIP and CIM as typical of the ICU because many ICU specialists might ignore ICUAW by perceiving the disorder as a complex problem in the acute stage of critically ill subjects that is not pertinent to their expertise. Of course, the involvement of the ICU specialist is essential and unique since he/she addresses ICUAW subjects in the early stages of disease occurrence, but different specialists should be involved in assessing this disorder and caring for patients suffering from ICUAW. A single specialist and medical field cannot embrace the long clinical course and multifaceted aspects of ICUAW subjects. Studies addressing risk factors, prevention, and therapeutic agents could be limited to intensivists, as is already the case. Conversely, in planning studies that investigate functional recovery, multiple specialists, such as neurologists and physiatrists, in addition to ICU physicians should be involved. A simple and not time-consuming EPS called the peroneal nerve test (PENT) has been proposed recently for responsive and unresponsive ICU subjects that can be performed by a clinical neurophysiology technician [30]. A neurologist could play a key role in examining questionable CIP and CIM electrophysiological pictures in depth, avoiding the risk of considering ICUAW as an occasional neurological disturbance that belongs only to the ICU field. Indeed, EPS and, in particular, dmCMAP should be performed by expert neurologists, who could contribute to differentiating the ICUAW types [15]. Ultrasound experts could contribute to diagnosing and monitoring the course of ICUAW types by new noninvasive and less time-consuming techniques. Indeed, novel ultrasound imaging techniques are promising to assess muscle changes that occur in critical illness, although they require confirmation [31, 32] and have limitations in differentiating between patients with and without ICUAW at relatively early stages in the disease course [33]. Partnerships with industries will be important to ameliorate and simplify these techniques to facilitate their use in clinical practice. It is important to admit subjects suffering from ICUAW to dedicated rehabilitative settings after ICU discharge since impairments and related disabilities might be due to ICUAW as well as to a combination of ICUAW and coexistent complex disorders, such as brain damage. A recent consensus of ICU experts has suggested that after ICU discharge, physical therapy interventions should include functional exercises, endurance training, strengthening exercises for limb and respiratory muscles, education on recovery, and a nutritional component [34]. In a dedicated rehabilitative setting, physiatrists could investigate rehabilitation interventions, provide proper functional outcome measures, and control visits. Different specialists might have difficulty in performing long-term follow-up after ICU discharge due to cultural hindrances, limited structures, and less familiarity in administrating functional measurements that are typical of rehabilitation expertise. Indeed, recovery evaluation, quality of life, and participation ascertainment require measurements and investigations that are in the remit of rehabilitation expertise. Furthermore, the patient might be more inclined to perform functional evaluations at rehabilitative facilities where the rehabilitation process can be carried out (Table 1).

**Table 1:** Main points

Studies by ICU specialists have permit understanding of several of the pathophysiological and clinical aspects of ICU-acquired weakness (ICUAW)
Despite important progress, the therapeutic strategies are unsatisfactory and issues such as functional outcomes and long-term recovery remain unclear
Subjects with a combination of acquired severe brain injury and ICUAW show reduced quality of life and greater disability compared to patients without ICUAW
Age, premorbid ICU condition, and functionality should be considered when evaluating ICUAW outcomes, but further aspects should also be considered, such as the ICUAW types, effect of the primary cause of ICU admission, role of each disorder in impairments and disability, and rehabilitation interventions
In planning studies that investigate functional recovery, multiple specialists, such as neurologists and physiatrists, in addition to ICU physicians should be involved
A multi-specialist approach might shed new light on areas of uncertainty and new insight into organizing better care pathways for ICUAW patients

## Conclusion

Although rehabilitative techniques have not been investigated, a dedicated rehabilitative setting could produce improvement in functional recovery in subjects with isolated ICUAW as well as in patients with ICUAW and coexistent disabling disorders. Given the protean aspects of ICUAW, a more strict collaboration as well as the participation of multiple specialists and experts are desirable either in planning future studies or in managing ICUAW subjects in clinical practice. A multi-specialist approach might shed new light on areas of uncertainty and new insight into organizing better care pathways for ICUAW patients.

## Abbreviations

CIM: Critical illness myopathy
CIP: Critical illness polyneuropathy
CIPNM: Critical illness polyneuropathy and myopathy
CMAP: Compound muscle action potential
dmCMAP: Direct muscle stimulation
EMG: Electromyography
EPS: Electrophysiological study
ICF: International Classification of Functioning, Disability and Health
ICU: Intensive care unit
ICUAW: Intensive care unit-acquired weakness
neCMAP: nerve stimulation
PENT: Peroneal nerve test
sABI: Severe acquired brain injury
